# Sex Differences in Heart Rate Variability and Vascular Function Following High-Intensity Interval Training in Young Adults

**DOI:** 10.5114/jhk/170964

**Published:** 2023-10-11

**Authors:** Myong-Won Seo, Tae-Young Park, HyunChul Jung

**Affiliations:** 1 Department of Exercise Science, David B. Falk College of Sport and Human Dynamics, Syracuse University, Syracuse, NY, USA.; 2 Department of Physical Education, Graduate School, Kyung Hee University, Yoingin-si, Gyeonggi-do, Republic of Korea.; 3 Sports Science Research Center, Kyung Hee University, Yongin-si, Gyeonggi-do, Republic of Korea.; 4 Department of Sports Coaching, College of Physical Education, Kyung Hee University, Yoinin-si, Gyeonggi-do, Republic of Korea.

**Keywords:** HIIT, autonomic nervous system, blood pressure, arterial stiffness

## Abstract

High-intensityintervaltraining (HIIT) issuperiortoothertrainingstrategies in both male andfemalehealthyindividuals. Understanding sex-specificdifferences in cardiac auto-regulation maycontributetothe optimal trainingstrategiesfor HIIT. The presentstudyaimedtoidentifysexdifferences in heart rate variability (HRV) andvascularfunctionfollowing HIIT in youngadults. Twenty-fourphysicallyactiveyoung male andfemaleadults (M: 12, F: 12, age: 19.5 yr, BMI: 22.1 kg·m^−2^) volunteeredtoparticipate in thestudy. Participantsperformed 10 boutsof HIIT including 20 s of high-intensitycycling at 115–130% W_max_followedby 100 s ofrecovery. The cardiac auto-regulationsincluding HRV andvascularfunctionweremeasured at five different time points. The R-R interval, rMSSD, and SDNN wererecoveredfaster in malesthan in females after 15 min of HIIT. Thereweresexdifferences in theautonomicnervoussystemwhereln LF andln HF activitiesalongwithsympathovagalbalance (ln LF/HF) weregreater in femalescomparedwithmalesimmediatelyand 15 min after HIIT. However, nosignificantdifferences in bloodpressureand brachial-ankle pulse wavevelocitywereobservedbetween male andfemaleparticipants. Overall, HRV was moreactivated in femalesthan in malesfollowing HIIT, but theacuteresponse in vascularfunction was not different betweensexes. In futurestudies, sex-specificadaptationsofcardiacautoregulationfollowingrepeated HIIT mayneedtobeperformed.

## Introduction

Participation in regular exercise programs plays an important role in improving physical performance and overall health (Thompson et al., 2003; Tian and Meng, 2019). High-intensity interval training (HIIT) has been introduced as one of the effective training regimes in improving cardiorespiratory performance compared with traditional training programs such as moderate-intensity continuous training (Wang et al., 2023; Way et al., 2020). Particularly, HIIT is recommended as a safe and time-efficient protocol for various populations such as athletes, older adults and patients with obesity and/or CVD (Dun et al., 2019; Rognmo et al., 2012). HIIT consists of repeated bouts of high-intensity movements alternated with low-intensity recovery periods, which elicit a positive response of cardiovascular autonomic function regardless of energy expenditure (Kessler et al., 2012; Kiviniemi et al., 2014). Many researchers have attempted to optimize HIIT protocols with respect to preventing and targeting various diseases (Hussain et al., 2016; Keech et al., 2020; Rudarli Nalcakan et al., 2021; Schmitz et al., 2018; Szumilewicz et al., 2022), but fewer studies have been focused on sex-specific differences in cardiovascular functions following HIIT in health young individuals.

There are various factors such as age, sex, individual health status, and fitness levels that have been considered when designing exercise programs. Understanding biological differences between males and females is particularly important to adequately prescribe HIIT regimes. In general, the cardiac size and stroke volume are smaller in women compared with men, but their cardiovascular homeostasis maintains a higher vagal tone in order to ensure adequate cardiac output (Beale et al., 2018). A recent meta-analysis also revealed that the autonomic nervous system (ANS) in women is characterized by greater parasympathetic activity and lower sympathetic inactivity compared to men, despite having a higher heart rate (Koenig and Thayer, 2016). In addition, women have higher vascular tone, shear stress, vascular compliance, and endothelium-independent dilation compared with men (Dengel et al., 2011; Pabbidi et al., 2018; Skaug et al., 2013). Nevertheless, the sex-specific response of cardiac auto-regulation and vascular function following HIIT remains unclear.

To the best of our knowledge, HIIT studies are primarily targeted at physically inactive or old adults (Alansare et al., 2018; Rebsamen et al., 2019; Songsorn et al., 2022; Tian and Meng, 2019; Gupta et al., 2022) despite the principal trainees being young adults. It is also important to understand the sex-specific differences of cardiac auto-regulation following HIIT, because these findings may provide valuable information to coaches and athletes for optimal HIIT strategies. Therefore, the aim of this study was to examine the sex-specific differences in heart rate variability (HRV) and vascular function following HIIT in healthy young adults.

## Methods

### Experimental Design

This study was performed to examine and compare sex differences of HRV and vascular function following a single bout of HIIT between physically active males and females. On the first visit, participants signed informed consent and then anthropometric measurements were performed along with body composition analysis. Next, the submaximal graded exercise test (GXT) was conducted on a cycle ergometer (Monark 828E, Sweden) to evaluate the individual exercise intensity. Participants performed cycling at 60 revolutions per minute (RPM) with 1 kilopond (kp), and the resistance increased by 0.5 kp every 2 min. The GXT was stopped when the participant could not continue with the increasing resistance of the cycle ergometer or could not maintain 60 RPM. Then, participants were fitted with a 3-axis ActiGraph GT9X Activity Monitor (GT9X, ActiGraph, USA) on the non-dominant wrist over seven days to measure their physical activity (PA) status. On the second visit, participants performed HIIT that consisted of 10 bouts of 20-s high-intensity cycling interspersed with 100-s rest intervals for a total of the 20-min exercise program with intensity ranging from 115 to 130 Watt_max_ (W_max_). HRV and vascular function were measured at five time points. In addition, participants were not allowed to drink any alcohol and caffeine for at least 24 hours prior to the second visit.

### Participants

A power analysis using the G*Power program 3.1.9.2 (Dusseldorf, Germany) was used to determine the sample size required to detect within-between factors for a repeated-measures ANOVA. With an estimated power of 0.80 and alpha of 0.05, a total sample of 24 was required to detect an effect size of 0.25. We continuously recruited participants who fully met the inclusion criteria for both groups until the sample size was sufficient. Twenty-four young adults (Male: 12, Female: 12, age [Mean ± SEM]: 19.5 ± 0.2 years, body height: 166.4 ± 1.6 cm, body mass: 61.5 ± 1.4 kg, BMI: 22.1 ± 0.3 kg·m^−2^) volunteered to participate in this study. Participants were enrolled if they met the following inclusion criteria: (a) young adulthood (defined as aged between 19 and 25 years), (b) 18.5 kg·m^−2^ ≤ BMI < 25 kg·m^−2^, (c) no history of any general disease, respective medication, and musculoskeletal injuries, (d) physically active. Participants’ PA was determined as greater than 150 min·weeks^−1^ of moderate-to-vigorous PA. The study was approved by the Institutional Review Board of the Kyung Hee University (KHGIRB-20-388, approval date: 7 October 2020). The study purpose, design, procedure, benefits, and potential risks were explained to participants and informed written consent was obtained from all of them.

### Anthropometric Measurements and Body Composition Analysis

Anthropometric variables, including body height and mass, were measured, and the BMI was calculated (body mass [kg] divided by body height squared [m^2^]). Body height and mass were measured in light clothing and without shoes using a portable standard stadiometer (Aluminum anthropometer, Samhwa instruments, Korea) and an electronic scale (150A CAS, South Korea), respectively. Body composition variables, including fat mass, body fat percentage, and lean body mass, were determined by dual X-ray absorptiometry (DEXA: QDR-4500W, USA).

### Heart Rate Variability and Vascular Function

Cardiac auto-regulation included heart rate variability (HRV) and vascular function. HRV was measured using a portable two-electrode, small device, and a non-invasive method (Actiwave cardio, CamNtech, UK). Prior to attachment of the device to an elastic chest belt at the participant’s fourth and fifth intercostal space, ECG (electrocardiogram) signals were set at a sampling rate and resolution of 1,024 Hz and 10-bit, respectively. Participants laid down on a soft bed in a dark and quiet room during the HRV recording. HRV was analyzed for an average of 5 min using fast fourier transform algorithms (FFT) by Kubios HRV Premium software (Kubios HRV premium 3.5.0, Kubios Oy, Finland). HRV was assessed for a total of 15 minutes for the baseline data analysis, and then the last 5 minutes were analyzed. After a single HIIT session, five additional HRV data analyses were taken within 65 min (i.e., 0–5 min, 15–20 min, 30–35 min, 45–50 min, and 60–65 min). HRV variables included both time domain and frequency domain variables. The time domain variables included the average heart rate (HR), the number of R-R intervals (IBI), the standard deviation of the R-R intervals (SDNN), and the square root of the mean squared differences of successive R-R intervals (rMSSD). The frequency domain variables were assessed, including low-frequency (LF, 0.04–0.15 Hz), high-frequency (HF, 0.15–0.45 Hz), and the LF/HF ratio. The vascular function, including bilateral brachial and ankle blood pressure (systolic blood pressure [SBP], diastolic blood pressure [DBP]) and bilateral brachial-ankle pulse wave velocity (ba-PWV), was assessed using a non-invasive portable vascular screening device (VP-1000, Omron, Japan), while participants were in the supine position in the same location as HRV monitoring. Pneumatic pressure cuffs were placed around the participant's bilateral upper arm and ankle, and two ECG monitoring electrodes were attached to the bilateral wrist. Additionally, a phonocardiogram sensor was placed over the left side of the 4^th^ rib to identify heart sounds. All measurements were assessed in duplicates, and the averaged values were used for further analysis. The vascular function was measured at five time points such as baseline, 5 min, 20 min, 35 min, 50 min, and 65 min after the HIIT session.

### High Intensity Interval Training

The HIIT program was performed using a Monark 894E Anaerobic ergometer bike (Monark 894E, Sweden). Before a single HIIT session began, participants performed a warm-up of at least 5 min at 60 RPM without any resistance. HIIT sessions consisted of 10 bouts of 20 s of high-intensity exercise followed by 100 s of rest intervals for a total of the 20-min exercise program with intensity ranging from 115 to 130 W_max_(applied kp; male: 3 kp, female: 2 kp). The intensity in the recovery phase was set at 60 RPM without any resistance. Exercise intensity was recorded by the Monark Anaerobic Wingate Software program (Monark Anaerobic Wingate Software, Sweden) during each bout of HIIT. Additionally, the rate of perceived exertion and the heart rate were monitored at every measurement time point after each bout. A certified strength and conditioning specialist (CSCS) designed the HIIT protocol and supervised all procedures.

### Statistical Analysis

All data analysis was performed using the SPSS software program 26 (SPSS, SPSS Inc, Chicago, IL, USA). The descriptive data were expressed as mean, standard error, and effect sizes. Repeated measures ANOVA was conducted to compare cardiac auto-regulation between males and females. If the results of repeated measures ANOVA were significant, the post-hoc test was performed using an independent *t*-test. The effect size was calculated as partial eta-squared (η^2^_p_; small ≥ 0.01, medium ≥ 0.06, large ≥ 0.14) and Cohen’s *d* (small ≥ 0.2, medium ≥ 0.5, large ≥ 0.8). The statistical significance level was set at 0.05.

## Results

The physical characteristics of male and female participants are presented in Table 1. Age and the BMI were not significantly different between male and female groups. Body height, body mass, and lean body mass were higher in males compared with females while females had a higher fat mass and body fat percentage compared with males.

**Table 1:** The results of basic characteristics of male and female participants.

Variables	Male	Female	Cohen’ *d*	*p* value
Age (year)	19.3 ± 0.2	19.6 ± 0.3	0.2	NS
Body height (cm)	171.8 ± 1.8	161.0 ± 1.5	1.9	0.001
Body mass (kg)	65.5 ± 1.5	57.4 ± 1.7	1.5	0.001
BMI (kg·m^−2^)	22.2 ± 0.4	22.1 ± 0.3	0.1	NS
Percentage body fat (%)	12.4 ± 1.0	24.3 ± 0.9	3.7	0.001
Fat mass (kg)	8.0 ± 0.7	13.8 ± 0.6	2.6	0.001
Lean mass (kg)	53.5 ± 1.3	40.4 ± 1.3	2.9	0.001

**Note:** data are mean ± SEM.

Table 2 presents sex-specific responses of HRV variables following HIIT. A significant interaction effect for the sexes by time point was shown for all variables, including the heart rate, R-R interval, rMSSD, SDNN, ln LF, ln HF, and ln LF/HF (all F(_1, 22_) ≥ 2.53, all *p* < 0.05). The heart rates and R-R interval were higher in female than male participants immediately after (heart rates; Cohen’s *d* = 0.85, R-R interval; Cohen’s *d* = 0.91) and 15 min after the HIIT session (heart rates; Cohen’s *d* = 1.06, R-R interval; Cohen’s *d* = 1.12). However, the rMSSD (Cohen’s *d* = 0.98) and SDNN (Cohen’s *d* = 1.29) were lower in female than male participants after 15 minutes of HIIT (*p* < 0.05). For ln LF and ln HF, females had lower values compared to males at immediately post exercise (ln LF; Cohen’s *d* = 1.00, ln HF; Cohen’s *d* = 1.16) and 15 minutes after the HIIT session (ln LF; Cohen’s *d* = 1.25, ln HF; Cohen’s *d* = 0.93) (all *p* < 0.05). Sympathovagal activity such as ln LF/HF was greater in males than females at the baseline (Cohen’s *d* = 1.50), but females showed higher ln LF/HF activity compared with males immediately after HIIT (Cohen’s *d* = 0.98, *p* < 0.05). Regarding vascular function, there were no significant interactions between sexes by time in all blood pressure and ba-PWV variables. The results are presented in Table 3.

**Table 2:** Changes in heart rate variability in male and female participants following high-intensity interval training

Variables	Sex	Baseline	Immediately post exercise	After 15 min	After 30 min	After 45 min	After 60 min	G × T Fvalue (η^2^_p_)
Heart rate (beat• min^−1^)	Male	68.9 ± 3.2	87.5 ± 4.1	78.1 ± 3.3	66.9 ± 3.3	64.5 ± 3.8	62.5 ± 2.9	8.46^***^ (0.28)
	Female	64.9 ± 1.9	98.1 ± 3.1	83.7 ± 3.0^#^	74.0 ± 3.3	71.7 ± 2.6	70.6 ± 2.7	
R-R interval (ms)	Male	904.8 ± 42.3	697.7 ± 28.5	850.3 ± 36.5	920.6 ± 43.3	964.2 ± 52.4	969.6 ± 39.3	6.06^***^ (0.22)
	Female	932.6 ± 26.8	618.8 ± 21.4^#^	727.4 ±26.4^#^	820.4 ± 26.1	848.7 ± 31.0	849.2 ± 41.9	
rMSSD (ms)	Male	47.4 ± 9.4	19.1 ± 5.9	31.8 ± 4.1	41.2 ± 5.4	49.9 ± 7.0	58.6 ± 7.6	3.92^**^ (0.15)
	Female	64.5 ± 5.5	9.3 ± 2.8	19.0 ± 3.4^#^	33.2 ± 4.7	40.7 ± 6.5	46.6 ± 7.4	
SDNN (ms)	Male	42.1 ± 5.9	21.8 ± 5.3	32.4 ± 3.1	37.7 ± 4.3	42.8 ± 4.4	58.3 ± 5.3	4.20^**^ (0.16)
	Female	51.3 ± 3.6	11.5 ± 2.2	19.5 ± 2.7^##^	30.9 ± 2.7	36.7 ± 4.1	46.4 ± 4.0	
ln LF	Male	6.5 ± 0.4	5.0 ± 0.3	6.1 ± 0.2	6.1 ± 0.3	6.4 ± 0.2	7.3 ± 0.2	2.53^*^ (0.10)
	Female	6.6 ± 0.2	3.9 ± 0.3^#^	5.6 ± 0.3^##^	5.6 ± 0.3	6.0 ± 0.3	6.8 ± 0.1	
ln HF	Male	6.4 ± 0.3	3.9 ± 0.4	5.6 ± 0.3	6.1 ± 0.3	6.5 ± 0.3	6.8 ± 0.3	8.26^***^ (0.33)
	Female	7.1 ± 0.2	2.7 ± 0.5^#^	4.4 ± 0.5^#^	5.6 ± 0.3	6.1 ± 0.3	6.5 ± 0.3	
ln LH/HF	Male	1.7 ± 0.3	3.2 ± 0.5	2.3 ± 0.6	1.6 ± 0.5	1.6 ± 0.6	2.1 ± 0.4	3.48^**^ (0.15)
	Female	0.7 ± 0.1^##^	6.7 ± 1.7^#^	2.6 ± 0.8	1.6 ± 0.5	1.6 ± 0.5	2.2 ± 0.6	

**Note:** R-R interval: the time elapsed between two successive R-waves of the QRS signal; SDNN: standard deviation of NN intervals; RMSSD: root mean square of successive RR interval differences: ln LF: average of 5-min segments of low-frequency power; ln HF: average of 5-min segments of high-frequency power; LF/HF: average of 5-min segments of the normalized low-/high-frequency ratio. G × T: group by time;^*^ Significant interaction effect, ^*^ p < 0.05, ^**^ p < 0.001, ^***^ p < 0.001, ^*^ Significant difference between groups, ^#^ p < 0.05, ^##^ p < 0.01.

**Table 3:** Changes in vascular function in males and females following high-intensity interval training.

Variables	Sex	Baseline	After 5 min	After 20 min	After 35 min	After 50 min	After 65 min	G × T F value (η^2^_p_)
Right-brachial SBP (mmHg)	Male	114.6 ± 2.4	117.4 ± 2.9	114.6 ± 2.4	111.7 ± 2.5	110.6 ± 2.4	111.8 ± 2.8	1.20 (0.05)
	Female	105.2 ± 1.8^#^	109.8 ± 2.3	108.2 ± 2.2	105.4 ± 2.5	105.3 ± 2.5	104.8 ± 2.8	
Right-brachial DBP (mmHg)	Male	61.7 ± 1.9	63.3 ± 3.3	62.8 ± 3.2	60.8 ± 2.7	56.3 ± 1.7	59.7 ± 2.1	1.81 (0.08)
	Female	59.6 ± 1.5	58.9 ± 1.5	58.1 ± 1.6	57.3 ± 1.7	58.0 ± 1.7	57.8 ± 2.2	
Left-brachial SBP (mmHg)	Male	113.3 ± 2.4	116.4 ± 3.3	113.3 ± 2.8	109.3 ± 2.6	109.6 ± 2.6	110.7 ± 2.8	0.64 (0.03)
	Female	104.0 ± 1.6	108.5 ± 2.1	106.5 ± 2.1	104.2 ± 2.0	101.5 ± 4.0	103.1 ± 2.7	
Left-brachial DBP (mmHg)	Male	61.7 ± 1.8	61.8 ± 2.4	58.3 ± 2.0	60.7 ± 2.7	56.7 ± 2.0	60.3 ± 2.3	1.41 (0.06)
	Female	58.4 ± 1.2	60.4 ± 2.1	57.9 ± 1.4	56.9 ± 1.5	58.2 ± 1.5	57.5 ± 2.5	
Right-ankle SBP (mmHg)	Male	130.9 ± 2.9	127.1 ± 4.0	129.8 ± 4.6	129.1 ± 3.6	128.8 ± 2.9	132.0 ± 3.6	0.27 (0.01)
	Female	119.4 ± 4.2	116.1 ± 2.9	119.3 ± 3.6	121.8 ± 3.9	120.3 ± 3.9	121.1 ± 4.1	
Right-ankle DBP (mmHg)	Male	64.4 ± 2.7	63.8 ± 2.5	62.2 ± 2.2	62.1 ± 3.4	61.4 ± 2.6	64.7 ± 2.8	0.62 (0.03)
	Female	61.3 ± 2.3	59.3 ± 2.0	59.7 ± 2.0	60.7 ± 2.7	61.2 ± 2.6	63.0 ± 3.1	
Left-ankle SBP (mmHg)	Male	127.8 ± 3.0	122.1 ± 3.1	127.1 ± 3.0	126.1 ± 2.9	125.9 ± 2.8	127.6 ± 3.4	0.12 (0.01)
	Female	121.7 ± 3.8	114.0 ± 3.0	119.8 ± 3.1	118.4 ± 3.9	119.3 ± 3.4	120.9 ± 3.9	
Left-ankle DBP (mmHg)	Male	63.3 ± 2.3	62.1 ± 2.3	61.3 ± 2.2	61.8 ± 3.8	59.3 ± 2.6	63.3 ± 2.1	0.37 (0.02)
	Female	61.1 ± 2.4	59.5 ± 1.7	59.9 ± 1.8	60.7 ± 2.2	60.3 ± 2.7	61.6 ± 2.8	
Right ba-PWV (m•s^−1^)	Male	1049.3 ± 39.8	1024.5 ± 40.7	1208.6± 39.5	1039.4 ± 35.3	1037.0 ± 50.8	1087.7 ± 35.9	0.94 (0.04)
	Female	966.0 ± 24.5	922.3 ± 32.6	911.9 ± 34.5	940.3 ± 32.6	950.3 ± 33.0	369.7 ± 25.7	
Left ba-PWV (m•s^−1^)	Male	1068.6 ± 40.4	1030.6 ± 37.7	1034.3 ± 39.7	1028.3 ± 30.7	1031.3 ± 49.3	1087.7 ± 35.9	0.53 (0.02)
	Female	969.7 ± 25.7	934.8 ± 32.2	921.1 ± 32.8	932.5 ± 33.4	961.6 ± 31.9	967.3 ± 30.6	

Note: SBP: systolic blood pressure; DBP: diastolic blood pressure; ba-PWV: brachial-ankle pulse wave velocity

**Supplementary Figure S1:**
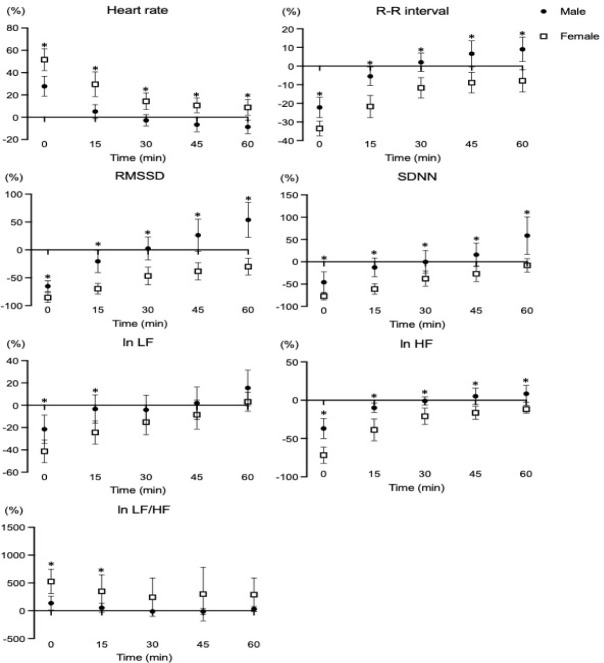
Percent changes of the time and frequency domain of HRV following a single HIIT session. LF: low frequency, HF: high frequency, ^*^p < 0.05: significant differences between groups

**Supplementary Figure S2:**
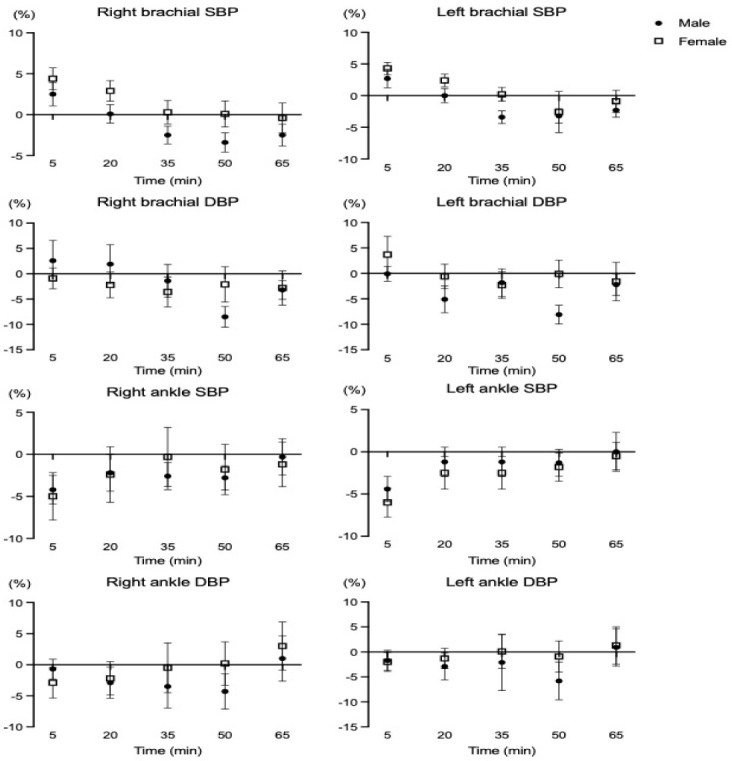
Percent changes of blood pressure of HRV following a single HIIT session. SBP: systolic blood pressure; DBP: systolic blood pressure

**Supplementary Figure S3:**
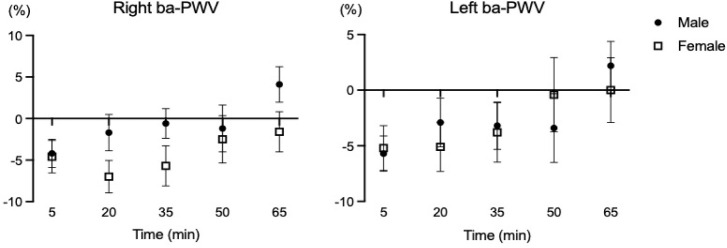
Percent changes of atrial stiffness following a single HIIT session. ba-PWV: brachial-ankle pulse wave velocity

## Discussion

The present study aimed to examine the sex-specific responses of cardiac auto-regulation following HIIT in young adults. Our findings showed that the time domain in males recovered faster than in females at least 15 min after HIIT. In addition, females elicited a higher ln LF/HF ratio compared with males immediately after HIIT. However, there were no significant sex-specific differences in any hemodynamic variables i.e., blood pressure and ba-PWV.

The changes of cardiac-autoregulation following acute exercise provide information on how to optimize exercise strategies and prescriptions with the goal to elicit better autonomic and cardiovascular function. The ANS is a key component in cardiacfunction and plays an important role in maintaining the balance and regulation of the cardiovascular system. HRV has been utilized as a non-invasive tool for identifying heart rate dynamics and potentially predicting cardiac health (Plaza-Florido et al., 2023). In the present study, the time and frequency domains were found to be greater, and the recovery was slower in females compared to males. The ANS stimulates sympathetic activity, regulates dynamics of the blood flow by decreasing parasympathetic and altering sympathetic innervation during exercise, and modulates the cardiovascular system's recovery following exercise to drive positive physiological adaptations (Borresen and Lambert, 2008). Although HRV is affected by the volume and intensity of the exercise program, the mechanisms underlying sex-specific differences in HRV are still unclear (Alemi et al., 2022;Koenig and Thayer, 2016; Moodithaya and Avadhany, 2012). The present study indicates that the HRV response to HIIT may vary between males and females, despite an identical HIIT protocol and intensity in healthy young individuals. In addition, the percent change in HRV in females returned to baseline status 60 minutes after HIIT except for the ln HF variable (refer to Supplemental Figure S1 in the supporting information). The ANS following HIIT may differ between sexes due to various physiological factors, including cardiac anatomy, hormonal levels, and cardiac auto-regulation. Wheatley et al. (2014) reported that females performed greater cardiac work compared with males under identical physical work demands, thus requiring compensation mechanisms for homeostasis of hemodynamic function. In addition, previous studies have demonstrated that sex hormones impact the autonomic nervous system. Although estrogen in females stimulates the parasympathetic nervous system in the follicular phase, rises in progesterone during ovulation stimulate the sympathetic nervous system, which increases the heart rate and decreases HRV (Stadler et al., 2019), whereas the male sex hormone, i.e., testosterone, regulates favorable alterations in autonomic function in the biological response to various stressors (i.e., physical and mental stress, environmental factors) (Ramesh et al., 2015). Therefore, sex hormones play an important role in cardiac modulation during the recovery phase after a single session of HIIT, and it may be that HRV in females is maintained at a higher level than in males after HIIT for cardiac protection despite using the identical HIIT volume and intensity. Nevertheless, it is important to note that these findings can vary depending on the age and the level of health-related physical fitness in females.

In the present study, the vascular function changes (i.e., brachial and ankle blood pressures, bilateral ba-PWV) were not different between males and females despite using the identical HIIT protocol. Supplementary analyses also showed no differences in percent changes of vascular function variables between male and female young adults (seeSupplementary data, Figure S2 and Figure S3). Doonan et al. (2013) demonstrated that there were no significant differences in carotid to femoral PWV, mean arterial pressure, and the subendocardial viability ratio between sexes after the maximal graded exercise with the Bruce protocol (Doonan et al., 2013). In a 12-week randomized intervention study describing the sex-specific adaptations of vascular function following two different exercise modes (Thomas et al., 2023), no significant sex-specific differences were found in blood pressure (i.e., SBP, DBP, MBP) after resistance and endurance training in healthy, physically active young adults (age: 25.6 years). It is possible that HRV is regulated to maintain vascular homeostasis and appropriate hemodynamic response to exercise (Pizzo et al., 2022). Nevertheless, sex-specific differences in vascular function following HIIT are not simple and affected by various factors such as genetics, age, cardiac anatomy, sex hormones status, etc.

The strengths of the present study include a) a first-time comparison study of cardiac auto-regulation response with an identical HIIT protocol and intensity between physically active young male and female adults; b) the use of non-invasive tools to monitor and assess the status of cardiac auto-regulation. However, there is a potential limitation that this study did not measure biochemical variables such as sex hormones (i.e., testosterone, estrogen), which could have provided insights into the mechanisms responsible for the confirmed association between sexes and acute cardiac auto-regulation response following HIIT. Further research is needed to investigate whether there are sex differences in HRV and vascular function after implementing various HIIT protocols and to include assessments of biochemical variables such as sex hormones.

## Conclusions

In summary, our study indicates that although both sympathetic and parasympathetic systems were more activated in females than in males following HIIT, acute response in vascular function was not different between sexes. Strength and conditioning coaches are able to understand sex differences of cardiac auto regulation following a single bout of HIIT and they can utilize our findings to design sex-specific HIIT programs. In future studies, sex-specific adaptations of cardiac auto regulationfollowing repeated HIIT should be evaluated.
